# Machine learning method for predicting line-shapes of Fano resonances induced by bound states in the continuum

**DOI:** 10.1038/s41598-025-16192-1

**Published:** 2025-08-25

**Authors:** V. S. Gerasimov, A. S. Kostyukov, A. E. Ershov, D. N. Maksimov, V. Kimberg, M. S. Molokeev, S. P. Polyutov

**Affiliations:** 1 International Research Center of Spectroscopy and Quantum Chemistry, Siberian Federal University, Krasnoyarsk, Russia 660041; 2 Institute of Computational Modelling SB RAS, Krasnoyarsk, Russia 660036; 3 Kirensky Institute of Physics, Federal Research Center KSC SB RAS, Krasnoyarsk, Russia 660036; 4 Theoretical Chemistry and Biology, KTH Royal Institute of Technology, 106 91 Stockholm, Sweden; 5 Laboratory of Theory and Optimization of Chemical and Technological Processes, University of Tyumen, Tyumen, Russia 625003; 6 Federal Siberian Research Clinical Centre under the Federal Medical Biological Agency, Krasnoyarsk, Russia 660037

**Keywords:** Metamaterials, Nanophotonics and plasmonics, Computational science

## Abstract

We consider resonances induced by symmetry protected bound states in the continuum in dielectric gratings with in-plane mirror symmetry. It is shown that the shape of the resonance in transmittance is controlled by two parameters in a generic formula which can be derived in the framework of the coupled mode theory. It is numerically demonstrated that the formula encompasses various line-shapes including asymmetric Fano, Lorentzian, and anti-Lorentzian resonances. It is confirmed that the transmittance zeros are always present even in the absence up-down symmetry. At the same time reflectance zeros are not generally present in the single mode approximation. It is found that the line-shapes of Fano resonances can be predicted to a good accuracy by the random forest machine learning method which outperforms the standard least square methods approximation in error by an order of magnitude in error with the training dataset size $N\approx 10^4$.

## Introduction

Optical bound states in the continuum (BICs) are source-free localized solutions of Maxwell’s equations which are spectrally embedded into the continuum of scattering states^1–5^. The optical BICs in dielectric metasurfaces have recently become an important instrument for resonant enhancement of light-matter interaction to be employed for resonant light absorption^6–10^, sensing^11,12^, harmonic generation^13–16^, and lasing^17–20^. Although BICs are not coupled to the incident light, breaking the system’s symmetry under variation of some control parameter^21,22^ results in the so-called quasi-BICs, i.e. long-lived resonant modes with the quality factor diverging to infinity on approach to the BIC in parametric space. This divergence is visible in the transmittance spectrum as a collapsing Fano resonance^23–28^ and simultaneously leads to electromagnetic field enhancement in the host metasurface^29,30^. This picture is generic in nanophotonics since high-quality resonant modes of any kind reveal themselves as sharp Fano resonances in the transmittance spectrum^31–34^. As shown in^35^ in the single resonant coupled mode approximation the Fano resonances can be described as a product of interference between two optical pathways, namely the resonant pathway due to the excitation of the resonant mode, and the direct or non-resonant pathway due to frequency independent background.

In this work we investigate the line shape of Fano resonances induced by symmetry protected optical BICs in dielectric gratings. The symmetry breaking leading to transformation of BICs to quasi-BICs is controlled by small deviation of the angle of incidence from the normal. Our goal is to analyse possible resonant line-shapes and find out weather the line-shapes can be predicted by machine learning methods using the geometric and optical properties as the input parameters. Our tool for describing the Fano resonance line-shape is the temporal coupled mode theory (TCMT)^35^. Nowadays, the TCMT is recognized as an efficient tool for describing the spectra of various phonic devices^36–43^ due to both universality and the clear physical picture it provides. It is worth mentioning that we are going to consider optical systems without up-down symmetry which can affect the line-shape of Fano resonances^38,44–48^. Optical and geometric parameters that give rise to BICs and quasi-BICs on metasurfaces are typically determined by numerically solving Maxwell’s equations. However, it is highly desirable to predict optical properties avoiding computationally expensive partial differential equations solutions for every possible parameter set. Instead, interpolation techniques based on existing data can offer a more efficient alternative. Nowadays, machine learning techniques have already been applied to various problems of nanophotonics^49–55^ including problems related to optical BICs^56–59^. Recently, the TCMT approach has been hybridized with neural networks^41,60,61^ for resonant response synthesis in photonic devices. Here we follow our previous work^62^ where we showed that the random forest machine learning method is capable of predicting the frequency of optical BICs in symmetric dielectric metasurfaces. We focus on BICs on metasurfaces structured as dielectric gratings^63,64^, which have recently drawn significant attention due to their applications in strong coupling^65^, sensors^66–69^, refractometry^70^, and absorption enhancement^71^. In what follows we revisit the TCMT in application to Fano resonances induced by symmetry protected BICs and apply the random forest method in combination with the TCMT to adress the line-shape prediction problem.

## TCMT equations

**Fig. 1:**
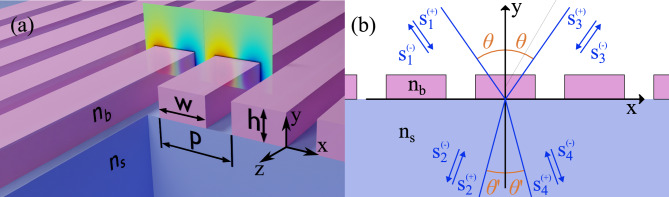
Metasurface in the form of a dielectric grating on a dielectric substrate. (**a**) Schematic of the metasurface with the electric field of the antisymmetric BIC mode. (**b**) Definition of the scattering channels.

As one can see in Fig. 1a the system under scrutiny is a ruled grating made of dielectric bars with refractive index $n_b$. The grating is placed on top of a dielectric substrate with refractive index $n_s$. The supestrate of the system is air with $n_0=1$. All geometric parameters, including the period *p*, the width *w*, and the height *h* are specified in Fig. 1a. In what follows we take $p=0.697~\upmu \text {m}$. In the system under consideration, the wavelength, linewidth, and all geometric parameters scale proportionally with the period *p*. As a result, the findings remain applicable to lattices with different periods. In this work we only consider TM-waves which propagate along the *x*-axis but not along the bars, so the scattering problem can be solved in the framework of 2D electrodynamics. The mode profile of a symmetry protected BIC is shown in the Fig. 1a in the form of the *z*-component of the electric field.

One can see in Fig. 1b that the scattering problem is described by a $4\times 4$ scattering matrix $\widehat{S}_{ {4}}$, which links the vectors of incident and outgoing amplitudes as follows

1 $$\begin{aligned} \mathbf{s}_{ (-)} =\widehat{S}_{ {4}} \mathbf{s}_{ (+)}, \end{aligned}$$

where the outgoing $\mathbf{s}^{ (-)}$ and incident $\mathbf{s}^{ (+)}$ vectors are given by

2 $$\begin{aligned} \mathbf{s}_{ {(\pm )}}= \left( \begin{array}{c} s_1^{ {(\pm )}} \\ s_2^{ {(\pm )}} \\ s_3^{ {(\pm )}} \\ s_4^{ {(\pm )}} \end{array} \right) . \end{aligned}$$

We assume that all dielectrics are lossless so the *S*-matrix is unitary $\widehat{S}_{ {4}}^{\dagger }\widehat{S}_{ {4}}=\widehat{{\mathbb {I}}}$. The system also possesses time-reversal symmetry therefore the *S*-matrix is symmetric $\widehat{S}_{ {4}}^{\intercal }=\widehat{S}_{ {4}}$. Importantly the matrix $\widehat{S}_{ {(4)}}$ must be of the block form

3 $$\begin{aligned} \widehat{S}_{ {4}}= \left( \begin{array}{cc} 0 & \widehat{S}_{ {2}} \\ \widehat{S}_{ {2}}^{\intercal } & 0 \end{array} \right) \end{aligned}$$

to ensure the momentum conservation in scattering from the metasurface^38^, where $\widehat{S}_{ {2}}$ is a $2\times 2$ unitary matrix. Since the system has the $\sigma _v$ mirror symmetry the problem can be reduced to finding $\widehat{S}_2$ that is a symmetric matrix as shown in Supplemental Document 1. Without a loss of generality we can focus on the left-going waves, so that

4 $$\begin{aligned} \left( \begin{array}{c} s_{1}^{ (-)} \\ s_{2}^{ (-)} \\ \end{array} \right) =\widehat{S}_2 \left( \begin{array}{c} s_{3}^{ (+)} \\ s_{4}^{ (+)} \\ \end{array} \right) . \end{aligned}$$

According to^35^ the TCMT equations describing single-mode scattering take the following form

5 $$\begin{aligned}&\frac{d a(t)}{d t}=-(i\omega _0+\gamma )a(t)+{\varvec{\kappa }}^{\intercal } \mathbf{s^{ (+)}}(t), \nonumber \\&\mathbf{s}^{ (-)}(t)=\widehat{C} \mathbf{s}^{ (+)}(t)+ a(t)\mathbf{d}, \end{aligned}$$

where $\widehat{C}$ is the matrix of direct (non-resonant) process, $\omega _0$ is the resonance center frequency, $\gamma$ is the radiation decay rate, *a*(*t*) – the amplitude of the resonant eigenmode, ${\varvec{\kappa }}$ is the coupling vector and $\mathbf{d}$ is the decoupling vector. In what follows we assume that the system is illuminated by a monochromatic wave of frequency $\omega$, so all time-dependent quantities in Eq. (5) oscillate in time with the harmonic factor $e^{-i\omega t}$. Importantly, the parameters in the TCMT equations are not independent, but linked to each other due to constraints imposed by energy conservation, Lorentz reciprocity and time reversal symmetry^35,72^. As we have already mentioned, the energy conservation manifests itself in the unitarity of the S-matrix whereas the time-reversal symmetry forces the S-matrix to be symmetric. In this situation the parameters of the TCMT equations are known to satisfy the following three equations^35^

6 $$\begin{aligned}&2\gamma =\mathbf{d}^{\dagger } \mathbf{d}, \nonumber \\&{\varvec{\kappa }}=\mathbf{d}, \nonumber \\&\widehat{C} \mathbf{d}^{*}+\mathbf{d}=0. \end{aligned}$$

In our case both energy conservation and time-reversal are present, however, care is needed in application of the time reversal operation since it maps the left-going waves onto the right-going ones. In Supplemental Document 2 we show that the $2\times 2$ unitary and symmetry of $\widehat{S}_2$ lead to the same constraints for the coupling parameters as in Eq. (6).

Now we have to solve Eq. (6) for the decoupling vector. We set out from the most generic form of $\widehat{C}$, which is unitary and symmetric,

7 $$\begin{aligned} \widehat{C} =e^{i\phi }\left( \begin{array}{cc} \rho e^{-i\eta } & i\tau \\ i\tau & \rho e^{i\eta } \end{array} \right) , \ \rho =\sqrt{1-\tau ^2} \end{aligned}$$

with $\tau \in [-1,~1]$, where all parameters are real valued. The parameters $\rho$ and $\tau$ in Eq. (7) are the absolute values of the reflection and transmission amplitudes, respectively, while $\phi , \eta \in [0, \pi ]$ describe the phase of scattered waves. The number of parameters in Eq. (7) can be reduced by redefining the incident channels via a unitary transformation

8 $$\begin{aligned} \left( \begin{array}{c} {s}_1^{ (+)} \\ {s}_2^{ (+)} \end{array} \right) = \left( \begin{array}{cc} e^{i\phi _1} & 0 \\ 0 & e^{i\phi _2} \end{array} \right) \left( \begin{array}{c} \tilde{s}_1^{ (+)} \\ \tilde{s}_2^{ (+)} \end{array} \right) . \end{aligned}$$

To be consistent with the time-reversal symmetry the outgoing channels have to be transformed as follows

9 $$\begin{aligned} \left( \begin{array}{c} {s}_1^{ (-)} \\ {s}_2^{ (-)} \end{array} \right) = \left( \begin{array}{cc} e^{-i\phi _1} & 0 \\ 0 & e^{-i\phi _2} \end{array} \right) \left( \begin{array}{c} \tilde{s}_1^{ (-)} \\ \tilde{s}_2^{ (-)} \end{array} \right) . \end{aligned}$$

Then, by using Eqs. (8) and (9) together with Eq. (7) one arrives at a single parameter family of unitary symmetric matrices

10 $$\begin{aligned} \widehat{C} =\left( \begin{array}{cc} \rho & i\tau \\ i\tau & \rho \end{array} \right) , \ \ \rho =\sqrt{1-\tau ^2}, \ \ \tau \in [-1, 1], \end{aligned}$$

if one chooses

11 $$\begin{aligned} \phi _1=\frac{\eta -\phi }{2}, \ \phi _2=-\frac{\eta +\phi }{2} \end{aligned}$$

in Eq. (8). It can be easely checked that the above unitary transformation complies with Eq. (6). The unitary transformation in Eq. (8) can be thought of as a shifting the reference plane between the scattering domain and the outer space along the *y*-axis. This is always possible in the far-field where the solution is exhaustively described by the scattering channels. By using Eq. (10) in Eq. (6) one finds a single-parametric family of solutions for $\mathbf{d}$ as follows

12 $$\begin{aligned} \begin{aligned} \mathbf{d}=&\sqrt{\frac{\gamma }{(1+\rho )}} \left( \begin{array}{c} \tau \cos \alpha -i(1+\rho ) \sin \alpha \\ \tau \sin \alpha -i(1+\rho ) \cos \alpha \end{array} \right) , \\ \alpha \in&[-{\pi }/{2}, {\pi }/{2}], \end{aligned} \end{aligned}$$

The derivation details are presented in Supplemental Document 3.

After using ${\varvec{\kappa }}=\mathbf{d}$ in Eq. (S10) one finds the final solution for the *S*-matrix

13 $$\begin{aligned} \widehat{S}=\widehat{C}+\frac{\mathbf{d} \mathbf{d}^{\intercal }}{i(\omega _0-\omega )+\gamma }. \end{aligned}$$

The transmission coefficient independent of the direction of incidence is written as

14 $$\begin{aligned} T=\frac{[\tau (\omega _0-\omega )+ \rho \gamma \sin (2\alpha )]^2}{(\omega _0-\omega )^2+\gamma ^2}. \end{aligned}$$

If $\alpha =\pm \pi /4$ Eq. (14) limits to the well-known solution presented in^35^ for system with up-down symmetry. The transmettance spectrum Eq. (14) complies with the earlier result from^46^ on the fundamental bounds on decay rates in asymmetric single-mode optical resonators, where it was shown that the transmittance is bound to peak to unity only in symmetric resonators. Equation (14) can be applied not only for TM-modes on dielectric metasurfaces. It remains valid for any optical material given that there is an isolated resonance coupled to two scattering channels.

The system supports a symmetry protected BIC in the $\Gamma$-point. With variation of the angle of incidence $\theta$ in the vicinity of the $\Gamma$-point the BIC is transformed to a high-*Q* resonant mode with the resonant frequency $\omega _0$ and the decay rate $\gamma$ given by the following Taylor expansion

15 $$\begin{aligned}&\omega _0=\omega _{ \textrm{BIC}}+\kappa _{\omega }\theta ^2+{\mathscr {O}}(\theta ^4), \nonumber \\&\gamma =\kappa _{\gamma }\theta ^2+\mathscr {O}(\theta ^4). \end{aligned}$$

Upon using the Taylor expansion Eq. (15) in Eq. (14) one arrives at

16 $$\begin{aligned} T=\frac{[\tau (\omega _{{ \textrm{BIC}}}+\omega _0^{ {(2)}}\theta ^2-\omega )+ \rho \gamma ^{ {(2)}}\sin (2\alpha )\theta ^2]^2}{(\omega _{{ \textrm{BIC}}}+\omega _0^{ {(2)}}\theta ^2-\omega )^2+(\gamma ^{ {(2)}}\theta ^2)^2}+{{\mathscr {O}}}(\theta ^6), \end{aligned}$$

which gives the line-shape of the Fano resonance induced by a symmetry protected BIC.

## Dataset acquisition

Our goal is to predict the shapes of the BIC-induced Fano resonance in the system shown in Fig. 1. According to Eq. (14), besides the resonance center-frequency $\omega _0$ and the radiation decay rate $\gamma$, which are specified by dispersion of leaky band hosting the BIC, there are only two parameters characterizing the shape of the Fano resonance, namely $\alpha$ and $\tau$. Both parameters can be found by fitting the numerically computed transmittance spectrum at the incidence angle slightly different from normal. In this work we take $\theta =2 \ \textrm{deg}$. The radiation decay rate and the center-frequency dictate the position and the width of the Fano resonance, correspondingly. Therefore, for predicting the shape of the resonance we have to analyse how the four parameters $n_b,~n_s,~h$ and *w* affect the quantities of $\alpha$ and $\tau$. Here we address this problem by applying machine learning algorithm to the data set obtained by numerically solving Maxwell’s equation under variation of all four control parameters. The ranges of parameters are specified below

17 $$\begin{aligned} n_b\in [2, 5], \ n_s\in [1.5, 4], \ h\in [0.2p, 0.8p], \ w\in [0.2p, 0.8p]. \end{aligned}$$

Note that the line-shape of the resonance is the same after a simultaneous change of the signs of both $\alpha$ and $\tau$. Following our previous work^62^ we focus on the wavelengh range 1100–2000 nm. Thus, we specify the following ranges for the property parameters

18 $$\begin{aligned} \tau \in [-1, 1], \ g \in [0, 1], \ \ 2000~{\text {nm}}> \lambda> 1100~{\text {nm}}, \ \gamma >0, \end{aligned}$$

where $\lambda$ is the wavelength of the resonance $\lambda =2\pi c/\omega _0$ and

19 $$\begin{aligned} g=\sqrt{1-\tau ^2}\sin (2\alpha ). \end{aligned}$$

**Fig. 2:**
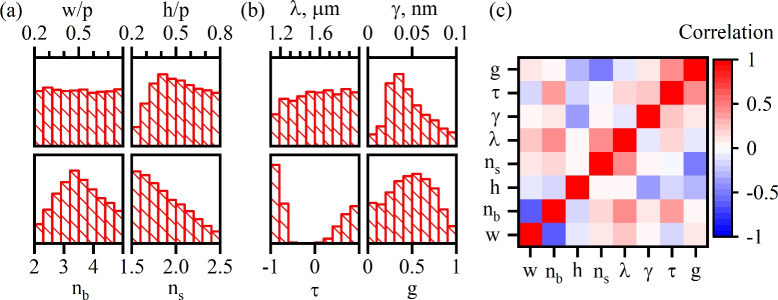
Distribution of (**a**) feature parameters and (**b**) calculated values of property parameters. (**c**) Correlation matrix between the feature and the property parameters.

To produce the dataset (see in Ref.^73^) we ran 100,000 numerical experiments of which 18,836 resulted in finding a symmetry protected BIC in the frequency range of interest. The simulations were preformed with application of the finite-element method (FEM) in COMSOL multiphysics package. The calculated values of the property parameters were extracted by least square fitting of Eq. (14) to the numerical data. The numerical experiments yielded values of four feature parameters (*h*, $n_b$, *w*, $n_s$) and four property parameters ($\lambda$, *g*, $\tau$, $\gamma$). In Fig. 2a we show the distribution of the feature parameters whereas the distribution of the property parameters is shown in Fig. 2b. One can see in Fig. 2a,b that most of the feature parameters and the BIC wavelength (property) exhibited almost uniform distributions. These uniform distributions suggest that the dataset encompasses representative cases. Additionally, the correlation matrix shown in Fig. 2c demonstrates the absence of linear relationships between the feature and property parameters, thereby justifying the application of machine learning methods.

## Random Forest method

In order to improve the precision of predicting the property parameters ($\tau$, *g*, $\lambda$, $\gamma$), we extended the dataset of feature parameters (*h*, $n_b$, *w*, $n_s$) incorporating their multiplication products, so the extended feature dataset also includes ($h^2$, $h\cdot n_b$, $h \cdot w$, $h\cdot n_s$, $n_b^2$, $n_b \cdot w$, $n_b \cdot n_s$, $w^2$, $w \cdot n_s$, $n_s^2$). For the prediction task, we utilized the random forest (RF) algorithm, a powerful ensemble method based on regression trees^74,75^. This approach involved constructing multiple decision trees by recursively partitioning the multidimensional predictor space. During the prediction phase, the RF model outputs the mode of the classes (for classification) or the mean average prediction (for regression) derived from the individual trees^76,77^. For the implementation of the RF model, we developed a Python script named RandomForest.py using the Python 3.6 programming language^78^. The script utilises the standard libraries, including *numpy, pandas, sklearn, matplotlib,* and *mpl_toolkits*. To account for the stochastic nature of the RF algorithm, we performed the 5-fold cross-validation test, aggregating the results to obtain an averaged performance and calculate the mean average error (MAE). Each iteration involved randomly splitting the data into two sets. One set comprising 70% of the total data was used for training the model. The remaining 30% of the data was used for testing. As a result, we constructed four distinct RF models, one for each property parameter ($\tau$, *g*, $\lambda$, $\gamma$).

Besides it predictive power, the RF algorithm can also quantify the importance of each feature parameter after training. This can be achieved by permuting the values of a selected feature within the training data and calculating the error on the perturbed dataset. The importance score for the feature is obtained by averaging the difference in error before and after permutation across all trees and subsequent normalization by the differences^79,80^. The features that yield higher values of this score are ranked as more important compared to features with lower values.

**Fig. 3:**
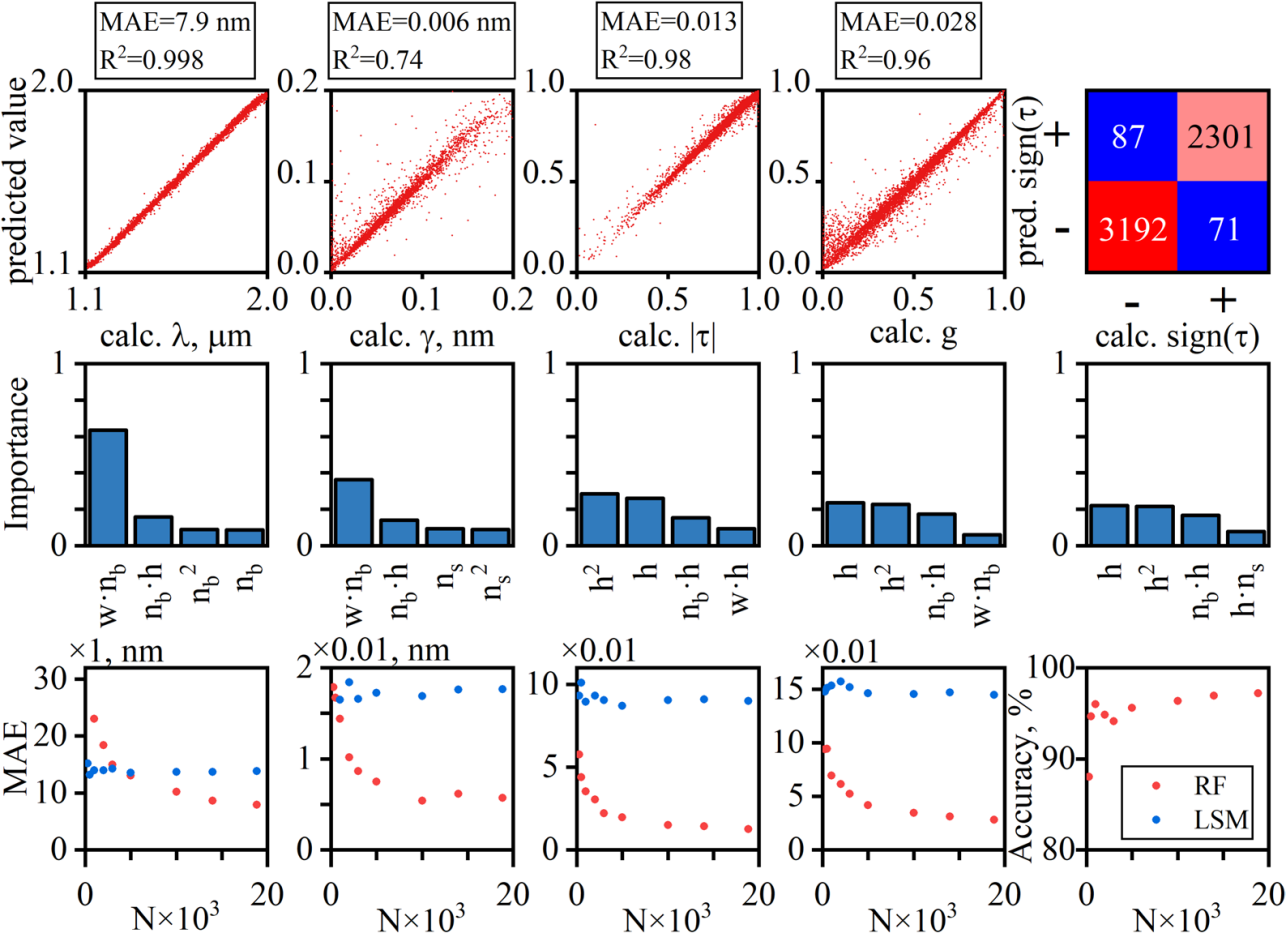
RF predictions for the four continuous property parameters $(\lambda , \gamma , |\tau |, g)$, and binary parameter $\textrm{sign}(\tau )$. The first row shows the calculated vs. predicted values of the property parameters. Note that for the binary parameters $\textrm{sign}(\tau )$ the results are visualized in the form of the confusing matrix. The second row shows the histogram plots of the four largest importance scores for the extended feature parameter set. The third row compares the performance of the RF method against the LSM depending on the size of the training dataset *N*.

Finally, in applying the RF method, it is found out that the algorithm fails to correctly predict the property parameter $\tau$ when its absolute value approaches unity. This is due to the structure of Eq. (14) in which the numerator becomes independent of the sign of $\tau$ when $\rho /\tau \ll 1$. To amend this difficulty we used (0,1) binary representation of $\textrm{sign}(\tau )$ to be predicted using the classification RF method. The quantity $|\tau |$ was used as the property parameter in application of the prediction RF instead of $\tau$. The results of application of the RF algorithm are collected in Fig. 3. In in Fig. 3 we plot the RF predicted versus calculated values of the four continuous property parameters ($|\tau |$, *g*, $\lambda$, $\gamma$). The plots are supplemented by histograms of the importance score of four the most important feature parameters, and by plots comparing the RF performance against the polynomial least square method (LSM). In the case of the binary parameter the performance is qualified by the confusion matrix.

## Results

We proceed to anylizing the data presented in Fig. 3. In the first row of Fig. 3 we plot the calculated versus the RF predicted values of the four continuous properties from the test data set. The last plot in the first row is the confusion matrix for $\textrm{sign}(\tau )$. The MAE and the coefficients of determination $R^2$ for all continuous properties are presented on the top of each plot. One can see that all properties are predicted to a good accuracy with exception of $\gamma$. This is due to the singular behaviour of the resonant linewidth in the spectral vicinity of a BIC. Namely, since $\gamma$ can be vanishingly small its value can change by orders in magnitude under small variation of angle $\theta$ near normal incidence, see Eq. (15). In the second row of Fig. 3 we plot the importance score of the four most important features from the extended data set in predicting all five property parameters. The data show that with the exception of the wavelength, which is predominantly determined by the optical path across the bar^62^, no other property is solely determined by a single parameter from the extended feature parameter set. The reader is referred to Ref.^62^ for a more detailed discussion on the prediction of $\lambda$. In the third row of Fig. 3 we compare the performance of RF against that of the polynomial LSM approximation in dependence on the size of the training data set. One can see that for all properties except the resonant wavelength, the RF significantly outperforms the LSM. Moreover, for the properties $|\tau |$ and *g*, which solely determine the resonant line-shape the MAE of the RF is $\approx$ one order of magnitude smaller that that of the LSM. Note that comparison against the least square method is impossible for the binary property $\mathrm{{sign}}(\tau )$, and therefor no LSM data are presented in the last plot of the third row in Fig. 3.

The data collected allows one to draw some conclusion on the shapes of Fano resonance induced by symmetry protected BIC. First of all the position of the resonance is dictated by wavelengh $\lambda$ corresponding to the resonant frequency $\omega _0$. This quantity can be accurately predicted by both the RF method and the LSM due to the fact that is is predominantly controlled by a single feature $w\cdot n_b$. The width of the resonance, albeit it is better predicted by the RF method than by the LSM, is the most difficult to predict due to the singular nature of the BIC. This does not, however, impose a difficulty since in any prefabricated set-up the resonant width is easily controlled by the angle of incidence. Finally, the line-shape of the resonance is controlled by $\tau$ and $\alpha$, both being efficiently predicted by the RF method. It is worth mentioning that according to the data from Fig. 2 the distribution of $\tau$ has the following momenta $\langle |\tau | \rangle =0.791$, and $\langle \tau ^2 \rangle =0.653$. It means that more often than not the resonance is observed on the background with transmission dominating over reflection. The first two momenta of the distribution of $\alpha$ are as follows $\langle \alpha \rangle =0.577$, and $\langle \alpha ^2 \rangle =0.376$. Remarkably, on average $\alpha$ is close to $\pi /4=0.785$ which corresponds to metasurfaces with up-down mirror symmetry. As it has been already mentioned the unit transmittance only occurs at $\alpha =\pi /4$. Thus, statistically the observed Fano resonances are likely to exhibit near-unit transmittance at the peak of Fano resonances.

**Fig. 4:**
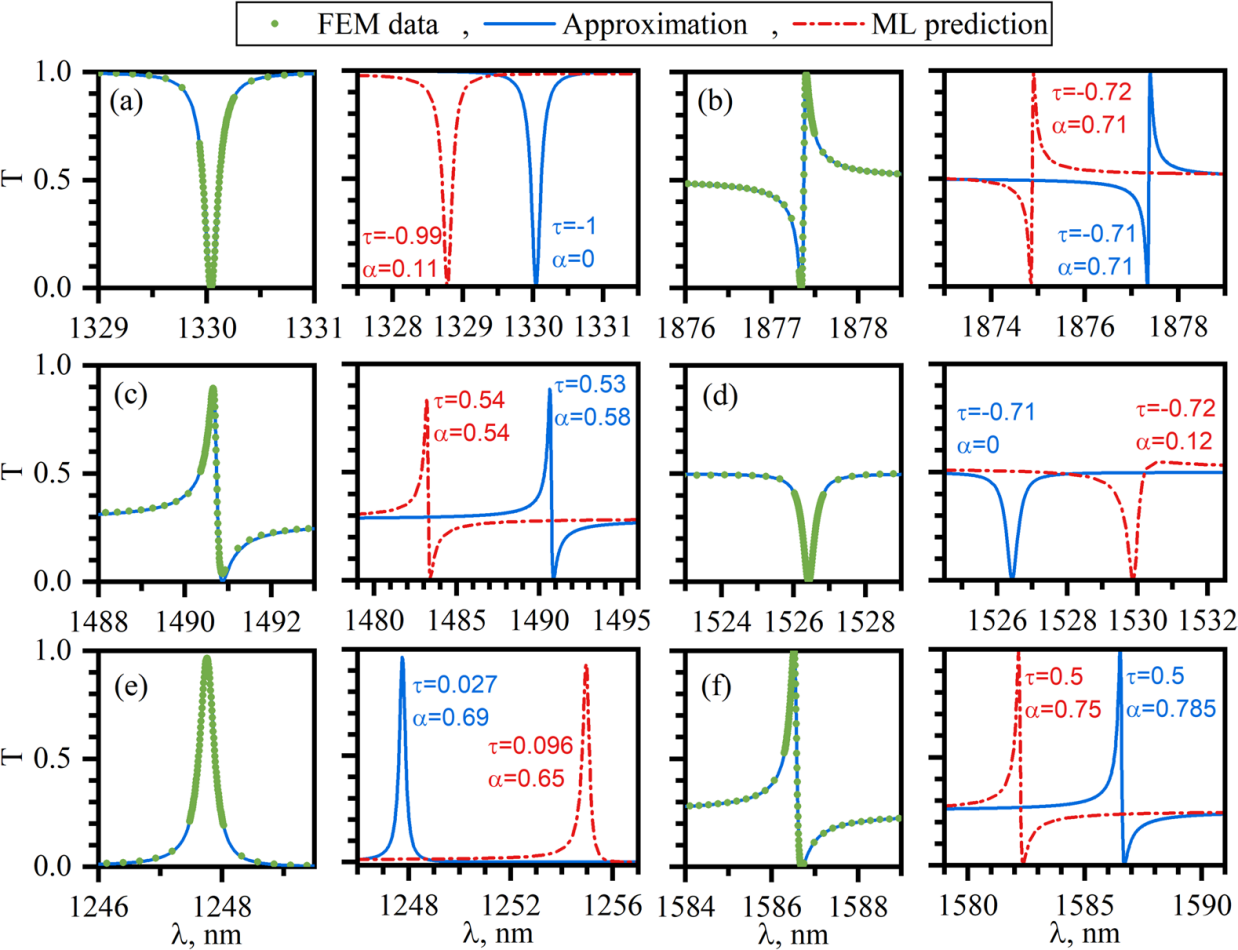
Fano resonances induced by symmetry protected BICs in dielectric gratings. FEM calculation data—green circles, Data approximation by Eq. (14)—solid blue lines, RF—predictions within the test dataset—dash-dot red lines. The numerically obtained and the RF predicted values of $\alpha$ and $\tau$ are shown in each sub-plot.

The profiles of Fano resonances are demonstrated in Fig. 4 where we plot six different line-shapes from the test dataset. For each case the FEM data are first compared against their approximation by Eq. (14) on the left panel of each subplot. On the right panel of each subplot we demonstrate the RF predicted line-shapes in comparison against Eq. (14). One can see that in each case the position of the RF predicted resonance is shifted with respect to the calculated one by distance bigger the the line-width of the resonance. This is due to vanishingly small line-width of the resonances with the average quality factor $Q = 8166$ in the spectral vicinity of a BIC across the dataset. Note that, although the according to Fig. 3 the resonant wavelength is predicted to a good accuracy, the RF fails to correctly position the resonance on the scale of its line-width. On the contrary the the RF predicted line-shapes of the resonance fit well to the calculated data. Note that different line-shapes are possible in the system under scrutiny including asymmetric Fano Fig. 4b,c,f, Lorentzian Fig. 4e, and anti-Lorentzian Fig. 4a,d line-shapes. Note that the transmittance always reaches zero at the dip of the resonances at the same time the numerically exact reflectance zeros are clearly absent in Fig. 4c,d. Finally, we note in passing that on approach to normal incidence the Fano resonance collapses and the transmittance becomes frequency independent with $T=\tau ^2$.

## Discussion

In this work we investigated line-shapes of the Fano resonances induced by symmetry protected bound states in the continuum in dielectric gratings. It is numerically demonstrated that the line-shapes are controlled by two parameters in Eq. (14) which encompasses various line-shapes including asymmetric Fano, Lorentzian, and anti-Lorentzian resonances. In full accordance with the previous studies^46,48,81^ it is confirmed that the transmittance zeros are always present even in the absence up-down symmetry. At the same time the reflectance zeros can only be approached accidentally when parameter $\alpha$ in Eq. (14) is close to $\pi /4$. It is found that the line-shapes of Fano resonances can be predicted to a good accuracy by the random forest machine learning methods which outperforms the standard least square methods approximation by an order of magnitude in error with the training dataset size $N\approx 10^4$. We speculate that the presented results may be useful for the design and synthesis of resonant responses in all-dielectric metasurfaces with one-dimensional periodicity. In the case of metasurfaces with two-dimensional periodic patterning, our approach would require generalization to account for polarization cross-talk. This represents an interesting direction for future research.

## Supplementary Information

Supplementary Information.

## Data Availability

Data supporting the findings of this study and Python script are available at GitLab: https://gitlab.com/nanoworld_ml/bic-ml.
