# Supplementary material for: Machine learning method for predicting line-shapes of Fano resonances induced by bound states in the continuum
Source: Sci Rep. 2025 Aug 25;15:31187. doi: 10.1038/s41598-025-16192-1 (PMC12379286; doi:10.1038/s41598-025-16192-1)
Supplement: Supplementary file 1 — Supplementary Information. [file 41598_2025_16192_MOESM1_ESM.pdf]

Machine learning method for predicting line-shapes of Fano resonances induced by bound states in the continuum

V. S. Gerasimov[1,2], A. S. Kostyukov[1], A. E. Ershov[1,2], D. N. Maksimov[1,3], V. Kimberg[4,*], M. S. Molokeev[3,5], and S. P. Polyutov[1,6]

[1]Siberian Federal University, International Research Center of Spectroscopy and Quantum Chemistry, Krasnoyarsk, 660041, Russia
[2]Institute of Computational Modelling SB RAS, Krasnoyarsk, 660036, Russia
[3]Kirensky Institute of Physics, Federal Research Center KSC SB RAS, Krasnoyarsk, 660036, Russia
[4]KTH Royal Institute of Technology, Theoretical Chemistry and Biology, Stockholm 106 91, Sweden
[5]University of Tyumen, Laboratory of Theory and Optimization of Chemical and Technological Processes, Tyumen, 625003, Russia
[6]Federal Siberian Research Clinical Centre under the Federal Medical Biological Agency, Krasnoyarsk, 660037, Russia
[*]kimberg@kth.se

1 S-matrix reduction

We start by rewriting Eq. (3) for the 4×4 S-matrix

$$\widehat{S}_4 = \begin{pmatrix} 0 & \widehat{S}_2 \\ \widehat{S}_2^{\intercal} & 0 \end{pmatrix} \tag{1}$$

The symmetry operation of the group C_2^z has a matrix representation

$$\widehat{P} = \begin{pmatrix} 0 & 0 & 1 & 0 \\ 0 & 0 & 0 & 1 \\ 1 & 0 & 0 & 0 \\ 0 & 1 & 0 & 0 \end{pmatrix}. \tag{2}$$

in the space of the incident/outgoing amplitude vectors. Now assuming that the channel functions are defined to be symmetric with respect to the mirror operation Eq. (2) one can state

$$\widehat{S}_4 = \widehat{P}^{-1}\widehat{S}_4\widehat{P}. \tag{3}$$

After substituting Eq. (1) to Eq. (3) one finds

$$\widehat{S}_2 = \widehat{S}_2^{\intercal}. \tag{4}$$

2 Derivation of Eq. (6)

The first line in Eq. (6) can be proven by the same method as suggested in[1]. First of all, we assume that the resonant eigenmode is normalized to carry a unit energy whereas the scattering carry a unit a energy per unit of time across the interface between the far-field and the scattering domain. Thus, the absence of incidence wave the energy conservation leads to

$$\frac{dE}{dt} = \frac{d|a|^2}{dt} = \mathbf{d}^{\dagger}\mathbf{d}|a|^2 \tag{5}$$

with E being the energy stored in the resonant mode. Given that the solution of the first line in Eq. (5) is

$$a(t) = a_0 e^{-(i\omega_0+\gamma)t} \tag{6}$$

we immediately have

$$\mathbf{d}^{\dagger}\mathbf{d} = 2\gamma. \tag{7}$$

The derivation of the other relationships in Eq. (6) is more complicated. We start from the time-harmonic substitution in Eq. (5) which leads to the time-stationary TCMT equations in the following form

$$[i(\omega_0-\omega)+\gamma]a = \kappa^{\mathsf{T}}\mathbf{s}^{(+)},$$
$$\mathbf{s}^{(-)} = \widehat{C}\mathbf{s}^{(+)} + \mathbf{d}a. \tag{8}$$

The solution of Eq.(8) can be written in the form of S-matrix

$$\widehat{S}_2(\omega) = \widehat{C} + \frac{\mathbf{d}\kappa^{\mathsf{T}}}{i(\omega_0-\omega)+\gamma}. \tag{9}$$

We notice that since $\widehat{S}_2(\infty) = \widehat{C}$ the matrix $\widehat{C}$ has the same symmetry and unitarity properties as $\widehat{S}_2(\omega)$. After applying $\widehat{S}_2^{-1} = \widehat{S}_2^{\dagger}$ we find

$$\widehat{C}^*\mathbf{d}\kappa^{\mathsf{T}}[-i(\omega_0-\omega)+\gamma] + \kappa^*\mathbf{d}^{\dagger}\widehat{C}[i(\omega_0-\omega)+\gamma]$$
$$+2\gamma\kappa^*\kappa^{\mathsf{T}} = 0. \tag{10}$$

By considering ω-dependent terms one finds that

$$\kappa^*\mathbf{d}^{\dagger}\widehat{C} = \widehat{C}^*\mathbf{d}\kappa^{\mathsf{T}}. \tag{11}$$

Applying the above equation in Eq. (10) and examining the terms independent of ω we write

$$\widehat{C}^*\mathbf{d}\kappa^{\mathsf{T}} = -\kappa^*\kappa^{\mathsf{T}} \tag{12}$$

Next, assuming that the coupling vector κ has at least one non-zero element we have

$$\widehat{C}\mathbf{d}^* = -\kappa. \tag{13}$$

Let us now utilize the symmetry of the scattering matrix. After applying $\widehat{S}_2 = \widehat{S}_2^{\mathsf{T}}$ in Eq. (9) we immediately have

$$\mathbf{d}\kappa^{\mathsf{T}} = \kappa\mathbf{d}^{\mathsf{T}}. \tag{14}$$

Multiplying the above equation from the left by $\mathbf{d}^{\dagger}$ one obtains

$$\kappa = \frac{\mathbf{d}^{\dagger}\kappa}{2\gamma}\mathbf{d}. \tag{15}$$

Alternatively, by multiplying Eq. (14) from the left by $\kappa^{\dagger}$ one has

$$\mathbf{d} = \frac{\kappa^{\dagger}\mathbf{d}}{\kappa^{\dagger}\kappa}\kappa. \tag{16}$$

The latter two equations combined result in

$$2\gamma\kappa^{\dagger}\kappa = |\mathbf{d}^{\dagger}\kappa|^2 \tag{17}$$

Next, by multiplying Eq. (13) from the left by its Hermitian adjoint one obtains

$$2\gamma = \kappa^{\dagger}\kappa \tag{18}$$

After analysing Eq. (15), Eq. (17), and Eq. (18) one can write

$$\mathbf{d} = e^{i\eta}\kappa,\ \eta \in [0, 2\pi). \tag{19}$$

Let us now give interpretation to the phase η. By recollecting that the resonant mode is normalized to carry a unit energy we immediately see that its normalization constant is defined up to an arbitrary phase factor. Thus, all the TCMT equations have to be invariant under the U(1) transformation

$$a = e^{i\alpha}a' \tag{20}$$

By plugging the above into Eq. (8) we find that it remains invariant, i.e. the same for the primed quantities, if the coupling and decoupling vectors are transformed as follows

$$\mathbf{d} = e^{-i\alpha}\mathbf{d}',$$
$$\kappa = e^{+i\alpha}\kappa'. \tag{21}$$

By choosing $\alpha = \eta/2$ one derives from Eq. (13) and Eq. (19)

$$\widehat{C}(\mathbf{d}')^* + \mathbf{d}' = 0,$$
$$\kappa' = \mathbf{d}'. \tag{22}$$

This equations are identical to the last two lines in Eq. (6).

3 Decoupling vector

We start with the third line in Eq. (6) that reads

$$\widehat{C}\mathbf{d}^* = -\mathbf{d}. \tag{23}$$

Vector $\mathbf{d}$ is in general parameterized by four independent real numbers

$$\mathbf{d} = \begin{pmatrix} a_{(\mathrm{r})} + ia_{(\mathrm{i})} \\ b_{(\mathrm{r})} + ib_{(\mathrm{i})} \end{pmatrix}. \tag{24}$$

Substituting the above into Eq. (23) one obtains a set of linear homogeneous equations of rank two

$$\begin{pmatrix} 1+\rho & 0 & 0 & \tau \\ 0 & 1-\rho & \tau & 0 \\ 0 & \tau & 1+\rho & 0 \\ \tau & 0 & 0 & 1-\rho \end{pmatrix} \begin{pmatrix} a_{(\mathrm{r})} \\ a_{(\mathrm{i})} \\ b_{(\mathrm{r})} \\ b_{(\mathrm{i})} \end{pmatrix} = 0, \tag{25}$$

which has the general two-parametric solution

$$\mathbf{d} = \begin{pmatrix} \tau b_{(\mathrm{i})} - i(1+\rho)a_{(\mathrm{i})} \\ \tau a_{(\mathrm{i})} - i(1+\rho)b_{(\mathrm{i})} \end{pmatrix}. \tag{26}$$

By recollecting that according to Eq. (6) $2\gamma = \mathbf{d}^\dagger\mathbf{d}$ one can find

$$\gamma = (1+\rho)(a_{(\mathrm{i})}^2 + b_{(\mathrm{i})}^2). \tag{27}$$

After combining Eq. (26) with Eq. (27) one arrives at the general solution of the form

$$\mathbf{d} = \sqrt{\frac{\gamma}{(1+\rho)}} \begin{pmatrix} \tau\cos\alpha - i(1+\rho)\sin\alpha \\ \tau\sin\alpha - i(1+\rho)\cos\alpha \end{pmatrix}, \ \alpha \in [-\pi/2, \pi/2], \tag{28}$$

where the range $[-\pi/2, \pi/2]$ is chosen since $\kappa = \mathbf{d}$ and, therefore, the sign of $\mathbf{d}$ is not important for the S-matrix Eq. (9).

References

1. Fan, S., Suh, W. & Joannopoulos, J. D. Temporal coupled-mode theory for the fano resonance in optical resonators. *J. Opt. Soc. Am. A* **20**, 569, DOI: 10.1364/josaa.20.000569 (2003).
